# *Enterococcus raffinosus*, *Enterococcus durans* and *Enterococcus avium* Isolated from a Tertiary Care Hospital in Romania—Retrospective Study and Brief Review

**DOI:** 10.3390/biology11040598

**Published:** 2022-04-14

**Authors:** Dan Alexandru Toc, Stanca Lucia Pandrea, Alexandru Botan, Razvan Marian Mihaila, Carmen Anca Costache, Ioana Alina Colosi, Lia Monica Junie

**Affiliations:** 1 Department of Microbiology, Iuliu Hatieganu University of Medicine and Pharmacy, 400012 Cluj-Napoca, Romania; stanca_lucia_pandrea@elearn.umfcluj.ro (S.L.P.); carmen_costache@elearn.umfcluj.ro (C.A.C.); icolosi@elearn.umfcluj.ro (I.A.C.); mjunie@elearn.umfcluj.ro (L.M.J.); 2 Regional Institute of Gastroenterology and Hepatology, 400000 Cluj-Napoca, Romania; 3 Cluj County Emergency Hospital, 400000 Cluj-Napoca, Romania; mihaila.razvan.marian@elearn.umfcluj.ro

**Keywords:** avium, casseliflavus, durans, enterococcus, gallinarum, other enterococci, raffinosus

## Abstract

**Simple Summary:**

*Enterococcus faecium* and *Enterococcus faecalis* are the most common species of *Enterococcus* spp. genus involved in human pathology. They are known for their increasing resistance to vancomycin, an antibiotic that blocks synthesis of Gram-positive bacteria’s cell wall. Other species of *Enterococcus* spp. (*E. casseliflavus*, *E. gallinarum*, *E. durans*, *E. avium*, *E. raffinosus*) are less common in human infections, and thus, their importance in the medical field is still uncertain. In this study, we analyzed the non-*faecalis* non-*faecium* Enterococci strains isolated from a tertiary care hospital in Romania for one year. Among a total of 658 *Enterococcus* isolates, 58 strains proved to be non-*faecalis* non-*faecium* Enterococci and met the inclusion criteria of our study. These species were isolated more frequently from mixed etiology infections with *E. coli* from the surgical ward. To put our results into perspective, a brief review of literature was performed in which we used 39 case reports involving non-*faecalis* non-*faecium* Enterococci. The emerging numbers of non-*faecalis* non-*faecium* Enterococci infections pose a danger to human health systems, due to their ability to easily acquire antibiotic resistance genes. To our knowledge, this study represents the first non-*faecalis* non-*faecium* Enterococci group analysis from Eastern Europe.

**Abstract:**

(1) Background: This paper aims to provide a description of non-faecalis non-faecium enterococci isolated from a tertiary care hospital in Romania and to briefly review the existing literature regarding the involvement of *Enterococcus raffinosus*, *Enterococcus durans* and *Enterococcus avium* in human infections and their antimicrobial resistance patterns; (2) Methods: We retrospectively analyzed all *Enteroccocus* species isolated from the “Prof. Dr. O. Fodor” Regional Institute of Gastroenterology and Hepatology from Cluj-Napoca during one year focusing on non-*faecalis* non-*faecium* Enterococci. A brief review of the literature was performed using case reports involving *Enterococcus raffinosus*, *Enterococcus durans* and *Enterococcus avium*; (3) Results: Only 58 out of 658 *Enteroccocus* isolates were non-*faecalis* non-*faecium* and met the inclusion criteria. These species were isolated more often (*p* < 0.05) from the surgical ward from mixed etiology infections with *E. coli*. In our review, we included 39 case reports involving *E. raffinosus*, *E. durans* and *E. avium*; (4) Conclusions: Isolation of non-*faecalis* non-*faecium* enterococci displays an emerging trend with crucial healthcare consequences. Based on the analysis of the case reports, *E. avium* seems to be involved more often in neurological infections, *E. durans* in endocarditis, while *E. raffinosus* displays a more heterogenous distribution.

## 1. Introduction

Based on rigorous criteria, the World Health Organization (WHO) elaborated in 2016 a list of antibiotic-resistant bacteria that should represent the top priority for global academic and corporate effort and funding into developing new antibiotics to fight the related infections. Among the Gram-positive bacteria, the highest priority was given to vancomycin-resistant *Enterococcus faecium*, followed closely by methicillin-resistant *Staphylococcus aureus* [1]. Although enterococci were known as commensals of the human gut as well as potential pathogens for decades, the publication of the WHO list led to a renewed interest in researching the *Enterococcus faecium (E. faecium)* and *Enterococcus faecalis (E. faecalis)* species, as they represent approximately 90% of the isolated enterococci in human infectious pathology [2,3].

With these two species being highly researched by the scientific community, it was noticed that other clinically significant species of the *Enterococcus* spp. genus were cultured from human biological specimens, notably *E. casseliflavus, E. gallinarum, E. durans, E. avium, E. raffinosus* and *E. mundtii* [4]. As these species are less frequent, they are collectively known as “non-*faecalis* non-*faecium* enterococci”, or simply “other enterococci (OE)”. These Enterococcus species were found in meat products and animal feces, carrying transmittable resistance genes for multiple antibiotics. It was also demonstrated that strains isolated from non-human sources can pass these genes to human strains, contributing to the overall burden of antibiotic-resistant infections [5].

The increasing resistance to vancomycin appears to be one of the greatest threats to public health, as this antibiotic is sometimes used as a last choice in infections with multiple resistant Gram-positive bacteria [6]. Vancomycin is a glycopeptide antibiotic that acts by binding to a key component of the Gram-positive bacteria’s cell wall, leading to a blockage in its synthesis and increasing its susceptibility to external factors.

The first vancomycin-resistant enterococci were discovered in 1988. Subsequently, it was found out that vancomycin resistance is achieved through a bacterium’s use of alternative cell wall synthesis pathways that are minimally influenced by the presence of the antibiotic in the environment [7]. Six phenotypes of vancomycin resistance have been described, namely *vanA*, *vanB*, *vanC*, *vanD*, *vanE* and *vanG*. Of these, *vanA* (resistance to both vancomycin and teicoplanin) and *vanB* (resistance to vancomycin but susceptibility to teicoplanin) are the most clinically significant ones, as they are coded by plasmid genes that may be transmitted horizontally (even to other genera, such as *S. aureus*). Meanwhile, *vanC* is coded by chromosomal genes, being described as intrinsic vancomycin resistance. *E. casseliflavus and E. gallinarum* are known to possess *vanC* non-transmittable genes [8].

Plasmid transmission of *vanA* resistance is a well-researched subject in *E. faecalis* and *E. faecium*. However, in recent years, nosocomial outbreaks of OE with the *vanA* vancomycin resistance phenotype were reported in the literature [9].

As little is known about the importance of OE isolates in human specimens and their antibiotic resistance profile, as well as the impact they have on overall morbidity and mortality, we propose a study that adds information to the general knowledge regarding their distribution among hospitalized patients, focusing on *Enterococcus raffinosus*, *Enterococcus durans* and *Enterococcus avium*.

## 2. Materials and Methods

This study was a longitudinal retrospective analysis of all enterococcus species isolated in the Regional Institute of Gastroenterology and Hepatology “Prof. Dr. O. Fodor” Cluj-Napoca, Romania, during a period of one year. We included the strains isolated from samples collected from the gastroenterology and hepatology department, surgery department and internal medicine department.

### 2.1. Strain Isolation, Identification and Antimicrobial Susceptibility Testing

Sample processing followed the hospital’s protocol, using specific media: sheep blood agar (bioMérieux, Marcy–l’ Étoile, France), Brilliance™ UTI Agar (Thermo Fisher Scientific, Waltham MA, USA). The strains were identified to the species level using a Vitek^®^ 2 Compact (bioMérieux, Marcy–l’ Étoile, France) GP card and with the antibiotic susceptibility performed using Vitek^®^ 2 Compact (bioMérieux, Marcy–l’ Étoile, France) AST P592 card.

### 2.2. Data Collection and Statistical Analysis

A database was generated using Microsoft Excel with the following variables: age, gender, diagnosis, sample type, number of hospitalization days, discharge status (recovered/deceased), *Enterococcus* species, associated bacteria or fungi if present, antimicrobial resistance profile and minimum inhibitory concentration (MIC). All information was gathered from the hospital’s electronic database.

To better understand the relation between the *Enterococcus* species, pathology and antimicrobial resistance, the samples were initially divided into three groups: *E. faecalis, E. faecium* and *OE*. The above-mentioned variables were presented distinctly for each group and an analysis of the differences between them was performed using the appropriate statistical tests. Regarding the antimicrobial resistance, the OE group was further divided into the *vanC* and non-*vanC* groups. The comparison between these groups was performed using the appropriate statistical tests for each variable.

For the OE species, the multiple antibiotic resistance (MAR) index was determined, representing the ratio between the number of antibiotics that an isolate is resistant to and the total number of antibiotics the organism is tested for. The MAR index was compared using the Kruskal–Wallis test for independent groups. A pair-wise comparison test between the MAR indexes of each species was also performed using the Bonferroni correction for multiple tests for the adjustment of the significance values. The statistical analysis was performed using IBM SPSS Statistics 26.0.

### 2.3. Brief Review Protocol

In order to provide a more comprehensive image regarding the involvement of *E. rafinosus, E. durans* and *E. avium* in human pathology, we performed a brief review. We searched relevant articles on PubMed, Cochrane library electronic database and Med Nar, up to 15 December 2020. We considered the following terms included in the studies title or abstract: “enterococcus”, “rafinosus”, “durans” and “avium” combined with the operator “AND” along with “human”, “infection” and “diagnostic”. The search was performed by two individual researchers and the results were confronted afterwards. We excluded studies written in languages other than English, French or Spanish.

## 3. Results

Although we initially found 658 isolates of *Enterococcus* involved in human infections, 319 (representing 48.48%) were excluded because they were not identified using the automated system Vitek^®^ 2 Compact (bioMérieux, Marcy-l’ Étoile, France). From the remaining isolates, 126 (37.16%) were *E. faecalis,* 155 (45.72%) were *E. faecium* and 58 (17.10%) were OE.

### 3.1. Descriptive Analysis of Enterococcus Species

A descriptive analysis was performed, focusing on the main characteristics of the patients, sample type, associated microorganisms and underlying conditions.

Table 1 contains the characteristics of the patients infected by the OE as well as a comparison between the species regarding age, gender, department, mortality and days of hospitalization. The mean age of the patients infected with OE was 64.33 years. More OE strains were isolated from male patients (*n* = 33) than female patients (*n* = 25). OE strains isolated from surgery/intensive care units (*n* = 43) were more common than those isolated from gastroenterology (*n* = 11) and internal medicine (*n* = 4) departments. Fatal infections with OE were declared in 29.3% of the cases.

OE strains were isolated from six urine samples, while three strains were isolated from urinary catheters. In total, 10 out of 58 OE strains were isolated from bile cultures, but only 7 of them from patients who underwent invasive gallbladder procedures. Puncture fluid cultures proved positive for 11 OE strains. The distribution of OE strains among other samples is presented in Table 2.

Mixed etiology infections involving one or two species of enterococci in association with other bacteria or fungi were further evaluated. Table 3 presents bacterial and fungal strains associated with OE. *E. faecium* was associated with OE in eight infections, while *E. faecalis* co-infections were proved in five cases. *Klebsiella* spp. proved to be the most common Gram-negative bacteria isolated from OE co-infections (*n* = 20). *Candida albicans* infections were also reported in nine cases of OE infections.

The main diagnoses of the patients presenting an infection with OE are given in Table 4 (number and percentage of patients for each disease included in the study). Cirrhosis and bloodstream infections proved to be the most common underlying conditions in patients with OE infection.

### 3.2. Antimicrobial Susceptibility Testing in Other Enterococci

**Figure 1:**
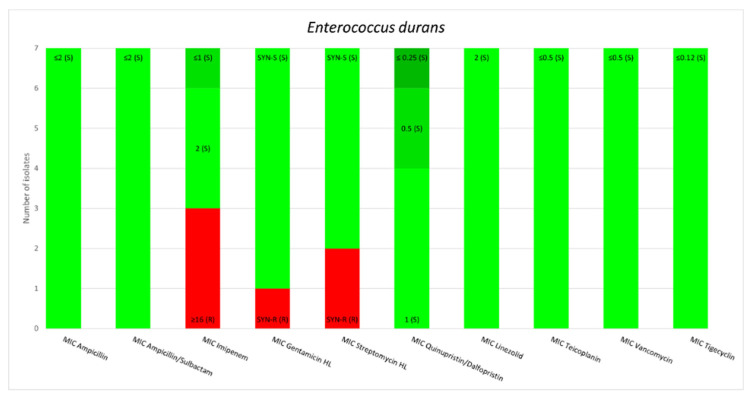

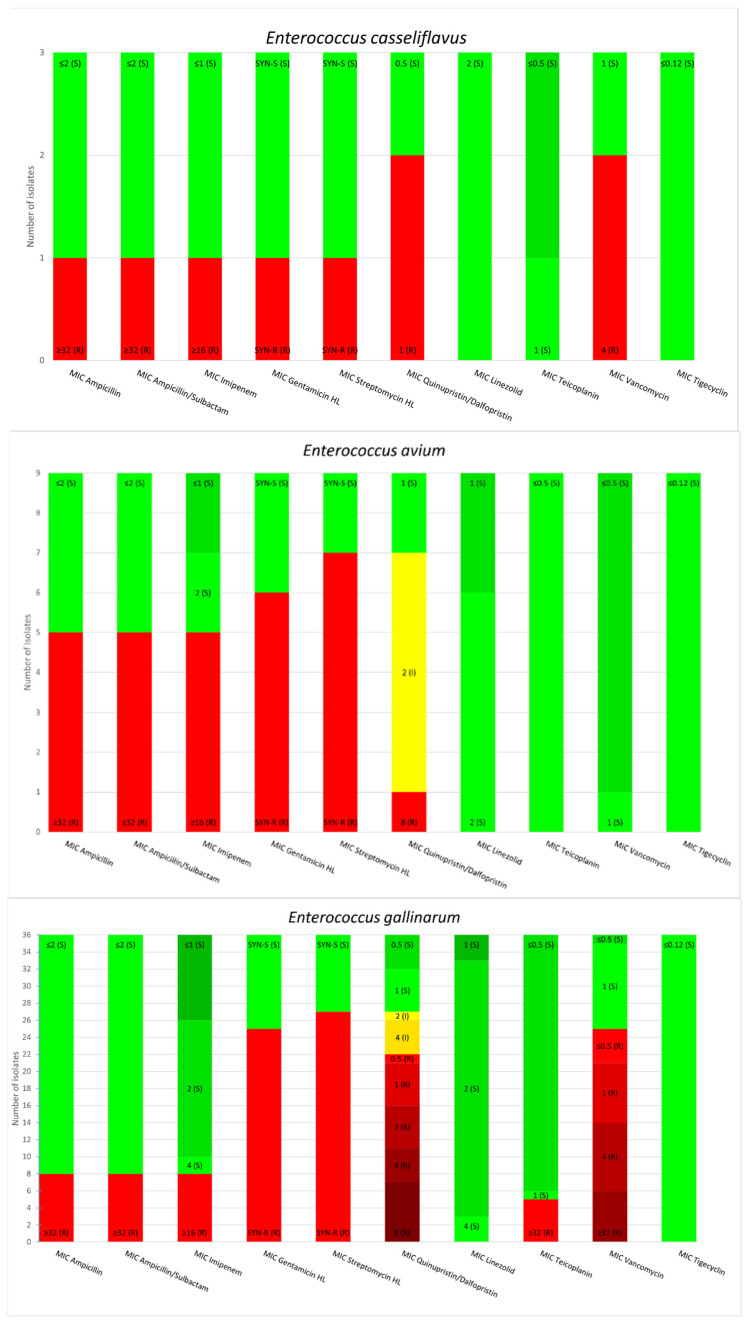

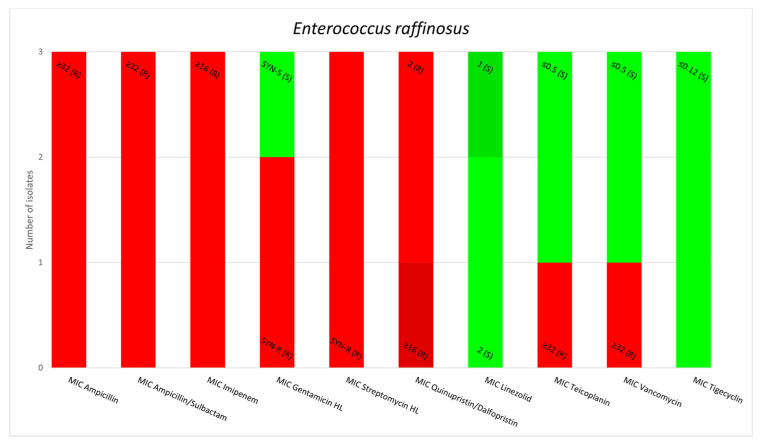
Minimum inhibitory concentrations patterns in *E. gallinarum*, *E. avium*, *E. raffinosus*, *E. casseliflavus* and *E. durans*.

*E. gallinarum* and *E. casseliflavus* are two species of OE that harbor the *vanC* gene; however, regarding the resistance to glycopeptides, the minimum inhibitory concentrations (MICs) of *E. gallinarum* for Teicoplanin equaled 1 mg/L for one strain and <0.5 mg/L for 30 strains. The MIC of *E. casseliflavus* for Teicoplanin equaled 1 mg/L for one strain and <0.5 mg/L for 2 strains. The MICs values for Tigecycline measured less than 0.12 mg/L for all tested strains of OE. Other antimicrobial susceptibility results for the OE in this study are presented in Figure 1.

### 3.3. Comparative Analysis of Other Enterococci

OE were organized based on their intrinsic resistance to glycopeptides into *vanC* and non-*vanC* enterococci. Both *vanC* and non-*vanC* strains were statistically significant isolated more frequently from surgical wards than from clinical wards (*p* = 0.012). Table 5 contains the differences related with different epidemiologic criteria and microbial association criteria between OE with *vanC* pattern and non-*vanC* from our study.

The comparison between OE, *E. faecalis* and *E. faecium* is presented in Table 6. OE are statistically significant isolated more frequently from polymicrobial infections than *E. faecalis* (*p* = 0.026) and *E. faecium* (*p* = 0.001). *E. coli* infections proved statistical significance in association with OE rather than *E. faecium* (*p* < 0.001).

**Figure 2:**
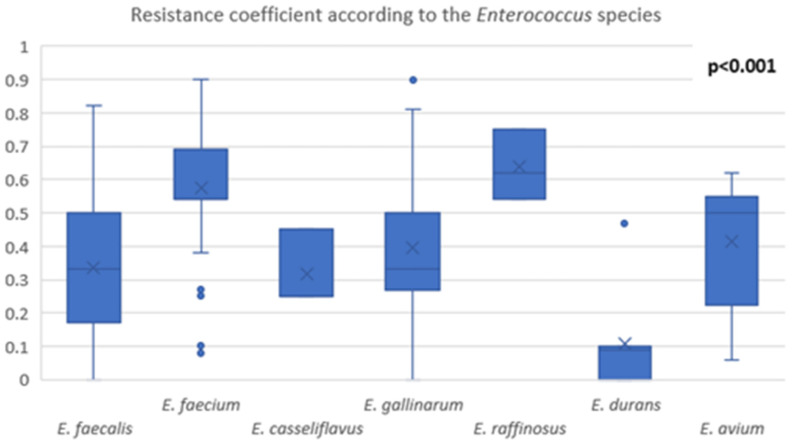
Comparison of MAR index among Enterococcus species.

The MAR index was studied for each strain of *Enterococcus*. *E*. *faecium* and *E. raffinosus* emerged as the most resistant species, with a MAR index above 0.6. A pair-wise comparison of the MAR indices and their statistical analysis are presented in Figure 2 and Table 7.

### 3.4. Brief Review of the Literature Concerning E. raffinosus, E. durans and E. avium

**Figure 3:**
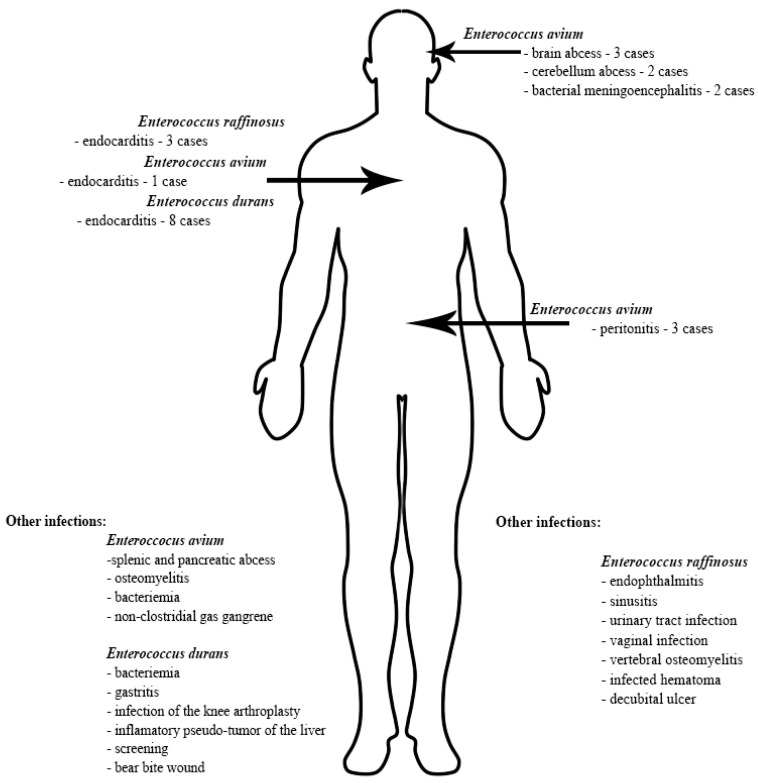
Infection localization of *E. avium*, *E. raffinosus* and *E. durans* [36,37,38,39,40,41,42,43,44,45,46,47,48,49,50,53,54,55,56,57,58,59,60,61,62,63,64,65,67,68,69,70,71,72,73,74,75].

Based on the protocol described above we included 39 case reports, 16 cases involving *E. avium*, 13 cases involving *E. durans* and 10 cases involving *E. raffinosus*. A summary of the case reports included in this brief review, organized based on the infection sites, is presented in Figure 3.

## 4. Discussion

Other Enterococci (OE) represent an often-forgotten group of human pathogens. Due to do the difficulty in isolating and diagnosing these bacteria, they remained for a long time in the shadow of more common pathogens from the *Enterococcus* genus, such as *E. faecalis* and *E. faecium*. Over the past decade, this dogma has changed drastically and once the automatic devices for isolation and detection became available worldwide, a surge of OE started to be reported. However, until recently, they remained an incidental finding without much knowledge regarding their pathogenicity [8,10].

Our work presents, to our knowledge, the first Eastern European extended report of OE isolated from human samples. We were able to isolate 58 strains belonging to 5 species of OE: *E. gallinarum* (36 strains), *E. caselliflavus* (3 strains), *E. avium* (9 strains), *E. durans* (7 strains) and *E. raffinosus* (3 strains).

Regarding the sample from where the strains were isolated, *E. gallinarum* was the only OE species isolated from the urine (nine strains), stool (two strains) and central venous catheter (one strain); moreover, *E. avium* was the only species isolated from the lower respiratory tract of a patient admitted to the Intensive Care Unit. Other products, such as bile, blood, pus, ascites and other fluids, presented a more prominent diversity regarding the isolates. However, regarding the bile culture, we observed that out of the twenty isolates of OE, fifteen were after an invasive procedure to the biliary tract. This was observed in other studies too, and currently, invasive procedures to the biliary tract represent a risk factor for infections with digestive tropism [11,12].

Another relevant observation represented the associations of OE with other pathogens, bacteria and fungi. OE are associated with a second pathogen in 84.48% of the cases, with *Klebsiella pneumoniae* representing the most common associated bacteria and *Candida albicans* the most common associated fungus. Thus, OE seem to be solely involved in human infections only in rare occasions. This may be another reason why these pathogens were isolated so rarely until recently and why their role in human infections is not clear [13,14,15,16].

The associated conditions of the patients with an isolated OE were cirrhosis, cholelithiasis, sepsis and malignancies (colon, stomach, pancreatic and bile ducts). These associations are not new and there are several reports that can further support these findings. Regarding the mechanism, some studies describe the involvement of vascular dilatation and permeation along with bacterial translocation like in cirrhosis. In colon cancer, *enterococcus* infections seem to be present due to alterations in the mucosa. However, none of the existing studies focus on OE so we are lacking information regarding their involvement, similarities and differences with *E. faecalis* and *E. faecium* [8,16,17,18,19,20,21,22].

Antimicrobial resistance patterns observed in our studies align with the existing information. None of the strains were resistant to linezolid and tigecycline, which remain two of the antibiotics used as a last resort in infections with VRE [8,23]. *E. raffinosus* strains were particularly resistant, displaying susceptibility only to linezolid, tigecycline, teicoplanin and vancomycin. This phenotype has the potential to become a real public health issue considering the ability of *E. raffinosus* to acquire circulating resistance genes, such as *vanA* or *vanB*, especially in some healthcare settings with high antimicrobial pressure, such as the intensive care units [9,24,25]. *E. avium* strains also presented a clinically relevant level of resistance similar to *E. raffinosus*. However, some of these strains were susceptible to ampicillin, ampicillin/sulbactam, imipenem, gentamicin, streptomycin and quinupristin/dalfopristin, which means that in contrast to *E. raffinosus*, infections caused by *E. avium* have more therapeutic options. The third OE species that does not display an intrinsic resistance phenotype to vancomycin, *E. durans*, has been found to be the most sensitive out of the three. The only resistances we observed in our study were to imipenem, gentamicin and streptomycin. All these findings are consistent with the information existing in literature and sustain the movement of developing new antibiotics [3,5,26,27].

To further evaluate the importance of OE, we divided them in two groups based on the intrinsic resistance to vancomycin. In this respect, the *vanC* group contains *E. casseliflavus* and *E. gallinarum,* while the non-*vanC* group contains *E. raffinosus*, *E. durans* and *E. avium*. We observed no significant difference between the two groups regarding age of the patients they infect, mortality, average length of hospitalization, association of *Klebsiella spp.* and *E. coli* and existing oncologic condition. However, non-*vanC* strains were isolated from the surgical ward significantly (*p* < 0.005) more often than *vanC* strains. We found no existing information in the literature to sustain this observation. We hypothesized that the antibiotic pressure from a surgical and critical care ward can select more resistant and diverse strains. Moreover, in our study, one additional factor that can explain these results was the hospital’s profile, being a gastroenterology and hepatology institute; most of the patients that required surgery involved an abdominal surgery, and thus, there is a higher chance of bacterial translocation [28,29].

To evaluate the implications of OE in human pathology, we compare them with the most recognized species of the genus, *E. faecalis* and *E. faecium*. There was no statistical difference between the groups concerning age, mortality, average length of hospitalization, association with *Klebsiella* spp. and *Candida* spp. and the surgical or clinical hospital ward. However, OE were isolated from mixed etiology microbial infections statistically more often than *E. faecalis* (*p* < 0.005) and *E. faecium* (*p* < 0.005). Additionally, the association of OE and *E. coli* was present statistically more often than *E. faecium* and *E. coli* (*p* < 0.005). These findings support the hypothesis that in our study, OE were isolated more often from the surgical ward due to the hospital’s profile (studies suggest the association of more than one microorganism in infections after abdominal surgery) [30,31].

The MAR index provides information regarding the antimicrobial resistance of each strain isolated. The statistical analysis performed revealed that *E. faecium* and *E. raffinosus* had significantly higher MAR indices than the other species. Although *E. faecium* is known to be highly resistant, our study showed that *E. raffinosus* has the potential to become a public health issue in the future regarding their antimicrobial resistance, despite the low prevalence of isolation of this pathogen in humans. However, these results must be considered with caution due to the low number of isolates. *E. durans* seems to be a less significant concern regarding the antimicrobial resistance, according to our results [32,33].

We further discuss the findings of our brief review focusing on *E. avium*, *E. durans* and *E. raffinosus* and some future perspectives.

### 4.1. Enterococcus avium

*E. avium* is a Gram-positive catalase negative *streptococcus*, commonly isolated from birds. In the past, *E. avium* was also known as *group Q streptococcus* [34]. Although it was described to cause bacteriemia and, thus, had the potential for other systemic infections, there are few case reports to date regarding the involvement of this pathogen in human infections [35].

In our review, we found only sixteen case reports describing the involvement of this bacterium in human pathology. Out of the sixteen cases, almost half of them presented a neurologic complication due to this bacterium: brain abscess or bacterial meningoencephalitis. We found three cases of brain abscess involving the temporal lobe and two cases of cerebellum abscess [36,37,38,39,40]. The association of chronic otitis media is common for all these cases. Although brain abscess represents less than 10% of all intracranial space-occupying lesions, due to the mortality rate described at about 25% in some studies and due to the unknown pathogenetic processes that may be involved, the association of *E. avium* and brain infections need to be further studied. Regarding the outcome of these cases, four of them survived and one patient died. The antimicrobial treatment in most of these cases was an association of a cephalosporin with vancomycin, metronidazole or amikacin. Escribano et al. described the association of linezolid and meropenem with a similar positive outcome [38].

Bacterial meningoencephalitis due to *E. avium* is described in only two cases, both with a positive outcome but without the association of chronic otitis media. This entity seems to involve a different pathogenetic process than the brain abscesses. Another relevant difference between brain abscess and bacterial meningoencephalitis relies around the age, with all the patients with brain abscess being less than 50 years old, while patients with bacterial meningoencephalitis were above 60 years old. However, this observation’s relevance is yet to be determined [41,42].

Regarding peritonitis, *E. avium* was involved in three out of the sixteen case reports [43,44,45]. Two cases involved patients around 60 years of age, with common risk factors being hypertension and peritoneal dialysis. Both patients were cured, but different treatment protocols were used. However, Ugur et al. described a pediatric case involving a female patient with several comorbidities which ultimately succumbed to the infection, despite the treatment with amikacin and vancomycin [45]. These observations suggest that *E. avium* may be more frequently involved than previously known in peritonitis in patients with peritoneal dialysis due to end stage kidney disease. Additionally, in cases with several other comorbidities or risk factors, *E. avium* seems to be able to produce a deadly infection.

Other cases reported *E. avium* as the causative agent in infections such as endocarditis, splenic and pancreatic abscess, osteomyelitis, bacteremia with a gastrointestinal starting point and even rare entities such as non-clostridial gas gangrene. All these findings presented an extraordinary heterogenicity concerning the infections caused by *E. avium* [46,47,48,49,50].

In the era of MALDI-TOF and other modern identification tools, we may be facing a real flood of new species and the real impact of *E. avium* on human health may become clearer.

### 4.2. Enterococcus durans

*E. durans* is part of the normal flora of the gastrointestinal tract. It is one of the rarest species of *Enterococcus* involved in human pathology, with few case reports existing since its discovery in 1935 by Sherman and Wing [51]. This is due to the low virulence, with few pathogenic factors described. Similar to other species of the genus enterococcus, *E. durans* is positive for the group D antigen in the Lancefield classification system. It is immobile and does not use mannitol as a source of energy, unlike other enterococcus species, namely *E. avium* and *E. raffinosus*. Other relevant biochemical characteristics are the positive reaction for the arginine test and negative reactions for the arabinose, pyruvate, raffinose and sorbose tests. This pattern of sugar metabolism makes *E. durans* difficult to diagnose in the microbiology laboratory if there is no automatic diagnostic equipment available, such as Vitek2Compact or MALDI-TOF [52].

In our review, we included thirteen case reports with *E. durans* as the etiologic agent. More than half, eight out of thirteen cases selected, were represented by infective endocarditis [53,54,55,56,57,58,59]. *E. durans* seems to be able to impair both the mitral and tricuspid valve as well as the aortic valve. It can affect the native valve and the prosthetic valve in the same manner. Concerning the severity, both cases that involved the mitral valve had a poor outcome, regardless of the treatment regime that was used [53,59]. Other endocarditis sites of infection seem to be associated with a better outcome. However, due to the low number of cases published concerning *E. durans* endocarditis, it is hazardous to draw more relevant observations and conclusions.

Other types of infection that were associated with *E. durans* were bacteremia, gastritis, infection of the knee arthroplasty, and inflammatory pseudo-tumor of the liver [60,61,62,63]. There is one published case where *E. durans* was isolated from a screening sample for ante natal identification of *Streptococcus agalactiae* and one case where it was isolated from a bear bite wound [64,65]. These might suggest the presence of *E. durans* in other significant sites of the microbiome than previously known and may also signify that *E. durans* can be isolated from a much wider variety of samples.

### 4.3. Enterococcus raffinosus

The most enigmatic out of the three species approached in this paper, *E. raffinosus*, is also the most recently isolated. It is tellurite- and arginine-negative, but mannitol-, sorbose-, arabinose-, raffinose- and pyruvate-positive. This biochemical profile along with the species being immobile make identification very difficult in laboratories that do not have an automatic detection method available [66].

Over the past two decades, case reports involving *E. raffinosus* in different human pathologies started to emerge. In our brief review, we included ten case reports of *E. raffinosus*. Unlike *E. avium* and *E. durans,* which seem to have a specific infection site, with *E. raffinosus*, there is more evident heterogenicity concerning the type of infections it can cause. However, regarding endocarditis, we found three case reports that have *E. raffinosus* as the etiologic agent [25,67,68]. Even if the antimicrobial protocol was different in these three cases, all of them had a positive outcome. The remaining seven case reports from our brief review describe infections at different sites of the human body: endophthalmitis, sinusitis, urinary tract infection, vaginal infection, vertebral osteomyelitis, infected hematoma and decubital ulcer. Most of these infections were treated with teicoplanin, a glycopeptide antibiotic, but the other antibiotic regimes seem to offer similar results [69,70,71,72,73,74,75].

Another relevant issue that concerned *E. raffinosus* is the ability of this bacteria to harbor the *vanA* gene that gives resistance to glycopeptides. There have been several studies recently that described actual outbreaks of *vanA E. raffinosus* in different types of facilities, making this OE extremely dangerous for medical environment and even for the public health [9,76]. Being difficult to diagnose without proper equipment and having the ability to harbor the *vanA* gene and possibly other resistance genes, *E. raffinosus* may prove itself a dangerous bacterium to combat. This represents further proof, if needed, that OE are an actual issue of public health, and their identification and antimicrobial testing should represent a priority for clinical microbiologists in the future.

### 4.4. Limitations

Regarding the limitations of our study, the retrospective nature of the study and the relatively low number of OE identified represent some of the most relevant aspects. Additionally, identification to the species level was not performed using MALDI-TOF or another molecular tool, since it was not available in our facility. Considering the brief review, the low number of the case reports included and their heterogenicity may affect the outcome.

### 4.5. Future Perspective

Based on the existing information, OE have the potential to become a real threat to the human health. Additionally, from an epidemiologic point of view, we might be facing outbreaks of OE harboring different ARG in the near future. However, recent papers are focusing on the probiotic potential of the *Enterococcus* genus [77]. This topic remains controversial due to the lack of proper information regarding some *Enterococcus* species, other than *E. faecalis* and *E. faecium*. While *E. durans* was analyzed from the probiotic potential point of view, *E. raffinosus and E. gallinarum* remain into question [77,78]. Still, the use of *Enterococcus* as a probiotic agent seems to have spiked global interest, but further studies are needed, specifically for OE, before a definitive conclusion is made [78].

## 5. Conclusions

Despite the paucity of published articles and isolated strains from human infections, there is proof that OE can be involved in severe infections with potentially a deadly outcome. The fact that they can harbor antibiotic resistance genes and can easily acquire further ones only adds to the danger that these *Enterococcus* species pose. This prompts an urgent need for further studies that can help us isolate, understand and treat the various infections that the OE group can cause.

To our knowledge, this paper provides the first analysis of the OE group from Eastern Europe and represents a milestone in the research of this niche.

OE have been in the spotlight recently concerning *E. gallinarum* and *E. casseliflavus* due to their vancomycin resistance via the chromosomal *vanC* gene. However, this paper provides comprehensive information regarding the involvement of *E. raffinosus, E. durans* and *E. avium* in human infections and their treatment.

## Figures and Tables

**Table 1:** Patient characteristics according to other Enterococci species.

		OE	*E. casseliflavus*	*E. gallinarum*	*E. raffinosus*	*E. durans*	*E. avium*	*p* Value
Number of cases (% of OE)		58 (100%)	3 (5.17%)	36 (62.06%)	3 (5.17%)	7 (12.06%)	9 (15.51%)	
Age (mean and standard deviation)		64.33 (12.70)	47(10.39)	63.61 (12.12)	76 (15.58%)	67.86 (11.05)	66.33 (12.10)	
Gender	Male	33	0	22	1	5	5	0.269
	Female	25	3	14	2	2	4	
Department	Surgery/ICU	43	2	23	2	7	9	0.332
	Gastroenterology	11	1	9	1	0	0	
	Internal Medicine	4	0	4	0	0	0	
Deceased (% of cases)		17 (29.3%)	0 (0%)	10 (27.77%)	1 (33.33%)	3 (42.85%)	3 (33.33%)	
Days of hospitalization (mean and standard deviation)		27.52 (26.86)	18 (10.39)	30.97 (30.99)	18 (4.58)	14.43 (8.22)	30.22 (23.97)	

Abbreviations: OE, other enterococci; ICU, intensive care unit.

**Table 2:** Distribution of other Enterococci among different samples.

Sample	*E. casseliflavus*	*E. gallinarum*	*E. raffinosus*	*E. durans*	*E. avium*	Total
Urine culture	-	9	-	-	-	9
Urine midstream	-	6	-	-	-	6
Urinary catheter	-	3	-	-	-	3
Stool culture	-	2	-	-	-	2
Bile culture	2	5	-	2	1	10
Invasive gallblader procedures	2	4	-	1	-	7
Central Venous catheter	-	1	-	-	-	1
Blood culture	-	1	1	1	-	3
Inferior Respiratory Tract	-	-	-	-	1	1
Pus	-	9	1	1	4	15
Puncture fluid	-	5	1	3	2	11
Others	1	4	-	-	1	6

**Table 3:** Association of different bacteria and fungi with other Enterococci (number of associations and percentage for each species described).

	*E. casseliflavus* (*n* = 3)	*E. gallinarum* (*n = 36*)	*E. raffinosus* (*n = 3*)	*E. durans* (*n = 7*)	*E. avium* (*n = 9*)	Total (*n* = 58)
*E. faecalis*	-	4 (11.11%)	-	1 (14.28%)	-	5
*E. faecium*	-	4 (11.11%)	2 (66.66%)	1 (14.28%)	1 (11.11%)	8
*Enterococcus* spp.	-	2 (5.55%)	-	-	1 (11.11%)	3
*Klebsiella* spp.	1 (33.33%)	15 (41.67%)	1 (33.33%)	2 (28.57%)	1 (11.11%)	20
*E. coli*	2 (66.66%)	6 (16,66%)	-	2 (28.57%)	5 (55.55%)	15
*Proteus* spp.	-	4 (11.11%)	-	-	2 (22.22%)	6
*Enterobacter* spp.	-	-	-	1 (14.28%)	-	1
*Morganella morgani*	-	1 (2.77%)	-	-	-	1
*Citrobacter* spp.	-	1 (2.77%)	-	-	1 (11.11%)	2
*Pseudomonas aeruginosa*	1 (33.33%)	5 (13.88%)	-	1 (14.28%)	1 (11.11%)	8
*Pseudomonas* spp.	-	1 (2.77%)	-	-	-	1
*Acinetobacter baumanii*	-	2 (5.55%)	-	-	2 (22.22%)	4
Coagulase-Negative Staphylococci	-	5 (13.88%)	-	-	1 (11.11%)	6
*Pantoea* spp.	-	1 (2.77%)	-	1 (14.28%)	-	2
*Providencia rettgeri*	-	1 (2.77%)	-	-	-	1
*Corynebacterium striatum*	-	1 (2.77%)	-	-	-	1
Associated fungi						
*Candida albicans*	-	4 (11.11%)	2 (66.66%)	-	3 (33.33%)	9
*Candida glabrata*	-	6 (16.66%)	-	-	1 (11.11%)	7

**Table 4:** Several underlying conditions associated with other Enteroccocus infections.

Underline Condition	*E. casseliflavus*	*E. gallinarum*	*E. raffinosus*	*E. durans*	*E. avium*	Total Number of Diseases
Cirrhosis	-	12 (100%)	-	-	-	12
Cholelithiasis	2 (25%)	3 (37.5%)	1 (12.5%)	2 (25%)	-	8
Bloodstream infections	-	3 (25%)	2 (16.66%)	2 (16.66%)	5 (41.66%)	12
Biliary Tract Malignancy	-	6 (85.71%)	-	1 (14.28%)	-	7
Colon cancer	1 (9.09%)	8 (72.72%)	-	1 (9.09%)	1 (9.09%)	11
Pancreatic cancer	-	3 (42.85%)	-	1 (14.28%)	3 (42.85%)	7
Gastric cancer	-	1 (100%)	-	-	-	1

**Table 5:** Comparison of *vanC* enterococci with non-*vanC* enterococci distribution.

	*vanC*	Non-*vanC*	*p*
Average age (SD)	62.33 (12.69)	68.42 (12.02)	0.087
Mortality/total	10/39 (25.64%)	7/19 (36.84%)	0.379
Average length of stay (SD)	29.97 (30.04)	22.47 (18.41)	0.323
Surgical ward/Clinical ward	25/14	18/1	0.012
*Klebsiella* spp. *	16/39 (41.02%)	4/19 (21.05%)	0.155
E. coli *	8/39 (20.51%)	7/19 (36.84%)	0.213
Oncologic/Non-Oncologic	19/20	7/12	0.417

Abbreviations: SD, standard deviation. * derived as co-culture.

**Table 6:** Comparison between other Enterococci and *E. faecalis* and *E. faecium*.

	OE (*n* = 58)	*E. faecalis* (*n* = 126)	OE-*E. faecalis* *p*	*E. faecium* (*n* = 155)	OE-*E. faecium* *p*
Average age (SD)	64.33 (12.70)	62.75 (13.70)	0.455	61.06 (14.65)	0.135
Mortality/Total	17/58 (29.31%)	36/126 (28.57%)	0.918	66/155 (42.58%)	0.077
Average length of stay (SD)	33.11 (35, 09)	27.52 (26, 86)	0.237	33.23 (31.65)	0.224
Surgical ward/Clinical ward	43/15	76/50	0.068	110/45	0.647
*Klebsiella* spp. *	20/58 (34.48%)	30/126 (23.80%)	0.130	38/155 (24.51%)	0.145
E. coli *	15/58 (25.86%)	25/126 (19.84%)	0.357	11/155 (7.09%)	<0.001
*Candida* spp. *	16/58 (27.58%)	23/126 (18.25%)	0.218	37/155	0.725
Associated agent	49/58	87/126	0.026	95/155	0.001

Abbreviations: OE, other Enterococcus; SD, standard deviation. * Derived as co-culture.

**Table 7:** Pairwise comparison of the MAR index of the Enterococcus species.

*p* Value Adjusted for Multiple Comparisons	*E. faecalis*	*E. faecium*	*E. casseliflavus*	*E. gallinarum*	*E. raffinosus*	*E. durans*	*E. avium*
*E. faecalis*	-						
*E. faecium*	<0.001	-					
*E. casseliflavus*	1.000	0.456	-				
*E. gallinarum*	1.000	<0.001	1.000	-			
*E. raffinosus*	0.226	1.000	0.862	0.898	-		
*E. durans*	0.644	<0.001	1.000	0.149	0.015	-	
*E. avium*	1.000	1.000	1.000	1.000	1.000	0.182	-

## Data Availability

Not applicable.
